# Zonal adjusted PSA density improves prostate cancer detection rates compared with PSA in Taiwanese males with PSA < 20 ng/ml

**DOI:** 10.1186/s12894-020-00717-z

**Published:** 2020-10-07

**Authors:** Tsung-Hsin Chang, Wun-Rong Lin, Wei-Kung Tsai, Pai-Kai Chiang, Marcelo Chen, Jen-Shu Tseng, Allen W. Chiu

**Affiliations:** 1 Department of Urology, MacKay Memorial Hospital, No. 92, Sec. 2, Zhongshan N. Rd., Zhongshan Dist, 10449 Taipei, Taiwan; 2 Mackay Medical College, No.46, Sec. 3, Zhongzheng Rd., Sanzhi Dist., Taipei, Taiwan; 3 Mackay Junior College of Medicine, Nursing, and Management, No.92, Shengjing Road, Beitou District, Taipei, 11272 Taiwan; 4 School of Medicine, National Yang-Ming University, No.145, Zhengzhou Rd., Datong Dist., Taipei, 10341 Taiwan

**Keywords:** Prostate cancer, PSA, Transition PSA

## Abstract

**Background:**

The current study aimed to compare the efficacy of transition zone PSA density (TZPSAD) with traditional PSA and PSA density (PSAD), for the diagnosis of prostate cancer (PCa) in Taiwanese males.

**Methods:**

Men with PSA between 4.0 and 20.0 ng/ml who underwent a transrectal ultrasound (TRUS) guided prostate biopsy between the studied period were retrospectively identified. The demographic data, PSAD and TZPSAD were calculated in all patients. Receiver operating characteristic (ROC) curves were used to analyze the accuracy of a positive PCa diagnosis.

**Results:**

The area under the ROC (AUC) was 0.615, 0.748 and 0.746 for PSA, PSAD and TZPSAD, respectively. The best cut-off of value for TZPSAD in predicting PCa in men with a PSA of 4.0–10.0 ng/ml was 0.367 ng/ml/ml with a sensitivity of 50% and a specificity of 77.5%. In men with a PSA of 10.1–20.0 ng/ml, the best cut-off value was 0.454 ng/ml, with a sensitivity of 74.8% and specificity of 70.9%.

**Conclusion:**

The use of TZPSAD can improve the efficiency and specificity of PSA for the diagnosis of PCa in Taiwanese men with PSA 4.0–20.0 ng/ml. TZPSAD efficiency was similar to PSAD but TZPSAD had better cancer specificity.

## Background

Prostate cancer (PCa) is one of the most common types of cancer among men worldwide, and the incidence rate of PCa has increased in recent years [1]. It has been reported to be the second most commonly diagnosed cancer and the sixth leading cause of cancer-related death among men worldwide [2–4]. This growing incidence may be due to the increasing trend for early detection across different countries [5]. Serum prostate-specific antigen (PSA) has been the most widely used marker for PCa screening and detection since its initial publication in 1987 [6]. However, PSA is more of an organ-specific marker than a cancer-specific marker, and several factors may cause a rise in PSA besides carcinoma, including, age, benign prostatic hyperplasia (BPH) and prostatitis. The increasing early detection of PCa may also increase the risk of over detection of indolent diseases and the risk of overtreatment, which may potentially expose men to harm from unnecessary procedures.

Therefore, multiple efforts have been made to improve the accuracy of PCa detection and avoid unnecessary procedures. Benson et al. [7] proposed a serum PSA/prostate volume ratio called prostate specific antigen density (PSAD) as an improved parameter for identifying PCa. Kalish et al. [8] were the first to study the use of transition zone prostate density (TZPSAD) as a more accurate predictor of PCa than PSAD is for PSA levels between 4.1 and 10.0 ng/ml. However, the prevalence of PCa differs among races and therefore the efficacy of different PCa detection strategies may also vary between races [9]. The use of TZPSAD in Asian populations has been previously reported and the results revealed improved detection rates [10–12].

To the best of our knowledge, no study has evaluated the efficacy of TZPSAD in Taiwanese men. Therefore, the present study was performed to assess whether using TZPSAD could improve the efficiency of PCa detection in Taiwanese males with a PSA level between 4.0 and 20.0 ng/ml.

## Methods

The current study retrospectively reviewed the medical records of patients who underwent a trans-rectal ultrasound (TRUS) guided biopsy of the prostate between October 2009 and December 2017 at our institution. A total of 1038 men with PSA levels between 4.0 and 20.0 ng/ml were identified. The exclusion criteria were patients with a previous history of prostate cancer, a prior history of prostate surgery, or those currently receiving hormonal regulation agents or taking 5-alpha reductase inhibitors.

All patients underwent imaging and laboratory studies prior to their TRUS biopsy. TRUS was performed in both transverse and sagittal views, using a BK medical Pro Focus ultrasound system type 2202, with a 5–10 MHz type 8808 prostate biplane transrectal probe. The anteroposterior (height) and transverse (width) dimensions were recorded in transverse planes and the superior-inferior (length) dimension was recorded in the sagittal plane. Total prostate volume (TPV) and transition zone volume (TZV) were calculated using the assumed ellipsoid prostate formula (volume = length × width × height × π/6) with the three linear dimensions measured from the TRUS. All total prostate volumes and transition zone volumes were calculated by three sonography technicians who had more than 20 years of experience. The 12-core TRUS prostate biopsy was performed by the same group of experienced urologists in one single medical center. PCa was defined as any positive biopsy cores reported by pathologists. The negative results were followed up according to NCCN and Taiwan Urology Association guidelines: PSA and DRE every 6–24 months.

A total of 755 men met the inclusion criteria and were included in the study; the demographic data, PSAD and TZPSAD were calculated for all patients. PSAD was calculated by dividing the patient’s PSA by their total prostate volume. TZPSAD was calculated by dividing their serum PSA concentration by the measured TZV. Diagnostic efficiency was calculated using the following formula: diagnostic efficiency (%) = sensitivity (%) × specificity (%)/100.

The present study, including its research protocol and data, were approved by the Mackay Memorial Hospital Institutional Review Board. All personal information was de-identified prior to the data analysis to ensure patient data confidentiality.

### Statistical analysis

All data were analyzed with and compared using a t-test and chi-squared test by IBM SPSS statistics software version 25.0 (IBM Inc., New York, USA). Statistical significance was set at p < 0.05. A receiver operating characteristic (ROC) curve was used to analyze the accuracy of PSA, PSAD and TZPSAD for the diagnosis of PCa.

## Results

The demographic data for the 755 studied patients is shown in Table 1. A total of 207 (27.4%) patients were diagnosed with PCa after their TRUS biopsy, while 548 (72.6%) patients were negative with a benign biopsy result. The mean age was 64.8 ± 9.26 years, the mean TPV was 50.59 ± 23.26 ml and the mean TZV was 31.95 ± 19.20 ml.

**Table 1:** Characteristics of the study cohort

Characteristic	PSA (ng/ml)		Total
	4.0–10.0	10.1–20.0	
No. of subjects (n)	465	290	755
Age (year)	62.8 ± 9.0	67.9 ± 8.67	64.8 ± 9.26
DRE positive	62	76	138
TPV	48.17 ± 20.25	54.46 ± 27.00	50.59 ± 23.26
TZV	29.90 ± 16.57	35.2 ± 23.58	31.95 ± 19.20
Biopsy positive	96	111	206
Positive detection rate (%)	20.6	38.2	27.2
DRE positive (n)	62	76	138
Gleason score			
≤ 6	68	64	132
7	21	19	40
8–10	7	27	34

Following a comparison between the cancer and non-cancer groups (Table 2), no significant age differences were observed. The PCa group showed a significantly higher PSA level (10.68 ± 4.05 vs. 9.09 ± 3.40, p < 0.001), a significantly higher positive finding following digital rectal exams (31.4% vs. 13.3%, p < 0.001), a significantly smaller TPV (39.21 ± 15.29 vs. 54.88 ± 24.30, p < 0.001), a significantly smaller TZV (23.15 ± 12.34 vs. 35.27 ± 20.20, p < 0.001), a significantly higher PSAD (0.31 ± 0.17 vs. 0.18 ± 0.09, p < 0.001) and a significantly higher TZPSAD (0.60 ± 0.49 vs. 0.33 ± 0.21, p < 0.001), compared with the non-PCa group.

**Table 2:** Clinical variables in PCa and non-PCa patients

	Prostate cancer	Non-PCa	p value
Total (n)	207	548	
4.0–10.0	96	369	
10.1–20.0	111	179	
Age (year)	67.20 ± 8.78	63.92 ± 9.28	0.732
DRE positive (n)	65	73	< 0.001
PSA (ng/ml)	10.68 ± 4.05	9.09 ± 3.40	< 0.001
TPV (ml)	39.21 ± 15.29	54.88 ± 24.30	< 0.001
TZV (ml)	23.15 ± 12.34	35.27 ± 20.20	< 0.001
PSAD (ng/ml/ml)	0.31 ± 0.17	0.18 ± 0.09	< 0.001
TZPSAD (ng/ml/ml)	0.60 ± 0.49	0.33 ± 0.21	< 0.001

*TPV* total prostate volume, *TZV* transition zone volume, *PSAD* PSA density, *TZPSAD* transition zone PSA density, *PCa* prostate cancer

**Fig. 1:**
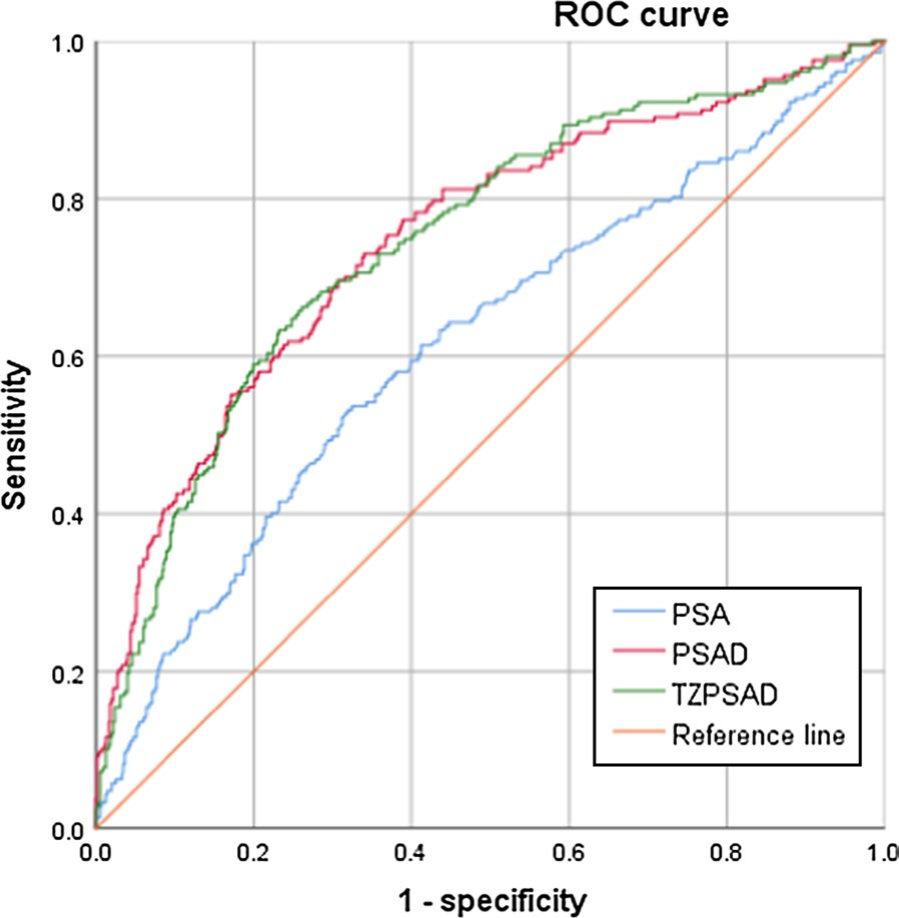
AUC of ROCs for overall PSA, PSAD and TZPSAD. The AUCs of the ROC for all patients were 0.615 for PSA, 0.748 for PSAD and 0.746 for TZPSAD

**Fig. 2:**
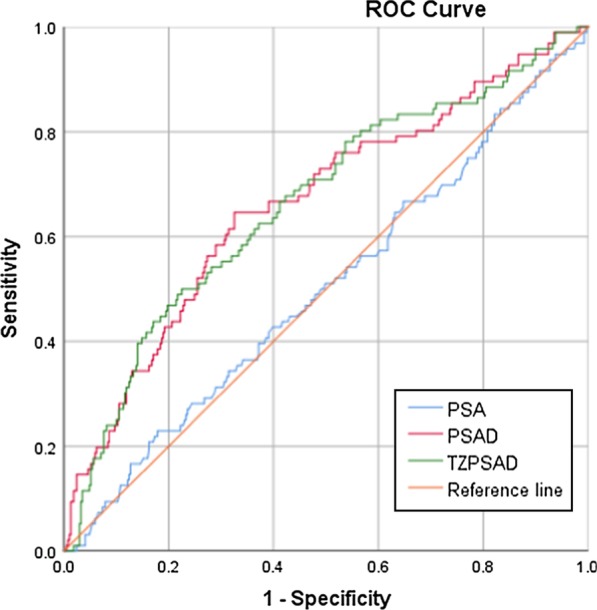
AUC of ROCs for PSA, PSAD and TZPSAD in patients with PSA level between 4.0–10.0 ng/ml. The AUCs for patients with a PSA level 4.0–10.0 ng/ml were 0.501 for PSA, 0.663 for PSAD and 0.663 for TZPSAD

**Fig. 3:**
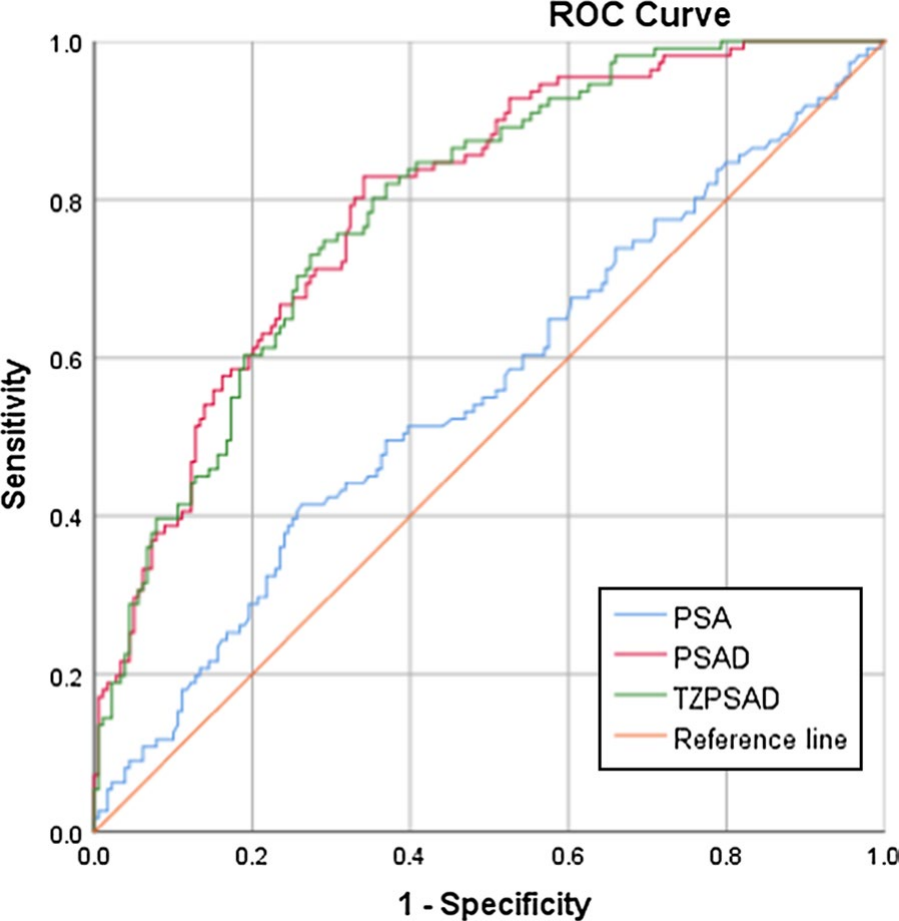
AUC of ROCs for PSA, PSAD and TZPSAD in patients with PSA level between 10.1–20.0 ng/ml. The AUCs for patients with a PSA level 10.1–20.0 ng/ml were 0.559 for PSA, 0.796 for PSAD and 0.792 for TZPSAD

ROC curves were performed for all patients and stratified by PSA level. PSA, PSAD and TZPSAD were analyzed. The area under the curve (AUC) of the ROCs are shown in Fig. 1. The AUCs of the ROC for all patients were 0.615 for PSA, 0.748 for PSAD and 0.746 for TZPSAD. The best cut-off point for PSAD was 0.198 ng/ml (sensitivity = 72.9%, specificity = 66.1%) and 0.403 ng/ml for TZPSAD (sensitivity = 63.3%, specificity = 76.8%). Patients were stratified by PSA level using the following groups: 4.0–10.0 ng/ml and 10.1–20.0 ng/ml (Table 3). The AUCs for patients with a PSA level 4.0–10.0 ng/ml were 0.501 for PSA, 0.663 for PSAD and 0.663 for TZPSAD (Fig. 2). The best cut-off value for PSAD and TZPSAD in predicting PCa in men with a PSA of 4.0–10.0 ng/ml were 0.174 ng/ml (sensitivity = 64.6% and specificity = 67.5%), and 0.367 ng/ml/ml (sensitivity = 50% and specificity = 77.5%), respectively. In men with a PSA 10.1–20.0 ng/ml, the AUCs for the ROCs were 0.796 and 0.792 for PSAD and TZPSAD, respectively (Fig. 3). The cut-off value for PSAD was 0.255 ng/ml/ml (sensitivity = 82.9% and specificity = 65.9%), and the cut-off value for TZPSAD in this group was 0.454 ng/ml/ml (sensitivity = 74.8% and specificity = 70.9%).

**Table 3:** Sensitivity, specificity, diagnostic efficiency and AUC under ROC

PSA level (ng/ml)	Parameter	Optimal cut-off	Sensitivity (%)	Specificity (%)	Efficiency (%)	Reducible biopsies (%)	AUC-ROC
4.0–20.0	PSA	10.085	53.1	68.1	36.2	68.2	0.615
	PSAD	0.198	72.9	66.1	48.1	55.2	0.748
	TZPSAD	0.404	63.3	76.8	48.6	65.6	0.746
4.0–10.0	PSA	8.590	22.9	82.1	18.8	81.0	0.501
	PSAD	0.174	64.6	67.5	43.6	60.8	0.663
	TZPSAD	0.368	50.0	77.5	38.8	71.8	0.663
10.0–20.0	PSA	14.535	41.4	73.7	30.5	67.9	0.559
	PSAD	0.255	82.9	65.9	54.6	47.2	0.796
	TZPSAD	0.454	74.8	70.9	53.0	53.4	0.792

## Discussion

The clinical manifestation of PCa is a spectrum that can range from a non-aggressive, slow-growing disease to a fast-growing, highly aggressive disease [1]. Recent guidelines and protocols focus on how to maximize the early detection of PCa that requires treatment and to minimize the overdiagnosis of unnecessary indolent diseases [13]. Serum PSA has been the most widely used marker for PCa screening and detection since its initial publication in 1987 [6]. However, the sensitivity and efficacy of PSA has been questioned. PSA screening may reduce PCa mortality but it is also associated with possible false-positive biopsy results that are accompanied by unnecessary biopsy-related complications [14], and the overdiagnosis of early stage disease [15]. Therefore, to avoid causing unnecessary harm, various methods have been introduced to try and increase the sensitivity of cancer detection, while maintaining good specificity, thus eliminating unnecessary biopsies.

Several PSA derivatives have been proven to be superior to PSA in predicting PCa. Benson et al. [7] first studied PSAD in 1992, which is serum PSA divided by prostate volume ratio, and showed that it was superior to PSA for identifying PCa. Kalish et al. [8] proposed the use of TZPSAD as an improved parameter for PCa instead of PSAD, for the traditional PSA “grey zone” (PSA levels between 4.1 and 10.0 ng/ml.). They hypothesized that since BPH mainly arose from the transition zone [16], PSA changes due to BPH should also result from the hypertrophied glands of the transition zone. Therefore, PSA formed by the outer zones (peripheral zone and central zone) should be relatively constant and less influenced in BPH patients. So, adjusting the PSA level with TZV and neglecting PSA changes from the outer glands should increase the ability of PSA to discriminate BPH from PCa. Several studies have shown that TZPSAD is a superior parameter compared with PSA alone [8, 10, 11, 17, 18].

PSA level and the prevalence of PCa differ among races and the efficacy of different PCa detection strategies may also vary for different races [9]. Tang et al. hypothesized that the true grey zone for PSA in Asian males should be higher than the traditional grey zone (4.1 ng/ml and 10.0 ng/ml.). They demonstrated that using TZPSAD can improve the efficiency of PSA in PCa diagnosis and avoid unnecessary prostatic biopsies in men with a PSA of both 4.0–10.0 and 10.1–20.0 ng/ml. The current study showed similar results to Tang et al. However, it is still unclear whether TZPSAD performed better than PSAD in predicting PCa. Despite some studies showing that TZPSAD outperformed PSAD in distinguishing PCa from BPH [8, 10, 19, 20], other investigators have claimed a different conclusion, saying that TZPSAD was not obviously superior to PSAD [21–23]. In the current study, similar AUCs and diagnostic efficiencies of TZPSAD and PSAD were found in all groups and there were not statistically significant differences. The two parameters showed equal efficacy and were both superior to PSA alone. However, the specificity of TZPSAD was better than PSAD, both overall and after PSA stratification, indicating that using TZPSAD as a method for PCa detection could improve the efficacy compared with traditional PSA and increase specificity, which may potentially avoid unnecessary biopsies when compared with PSAD.

There were several limitations to the current study that need to be addressed. (1) Its retrospective nature; (2) the accuracy of ultrasound measurements is operator dependent. Therefore, measurements by different sonographers may differ and influence the outcomes of TZPSAD. In the present study, all of the prostate volumes were measured by 3 very experienced staff to minimize this bias. (3) All the data and patients were collected from a single medical center. The strategies used for PCa diagnosis, including TRUS measurement and TRUS biopsy, may differ from other institutions, which may cause an inherent bias. Therefore, further studies should be conducted with a larger number of patients who are prospectively randomized, to minimize the bias.

## Conclusions

In this study, TZPSAD showed better PCa detection over serum PSA alone. However, the efficiency of TZPSAD was equal to PSAD in predicting PCa while TZPSAD had slightly better cancer specificity. Prostate volume derived TZPSAD estimate in Taiwanese men with a serum PSA level < 20 ng/ml outperformed PSA alone but does not allow for better risk-stratification than PSAD.

## Data Availability

Records and data pertaining to this study are in the patient’s secure medical records in Mackay Memorial Hospital and are available from the corresponding author on reasonable request.

## Abbreviations

PSA: Prostate specific antigen
TZPSA: Transition zone PSA
PSAD: PSA density
TZPSAD: Transition zone PSA density
PCa: Prostate cancer
TRUS: Transrectal ultrasound
BPH: Benign prostatic hyperplasia
